# Infective Endocarditis Due to *Kingella kingae*

**DOI:** 10.3390/microorganisms12010164

**Published:** 2024-01-13

**Authors:** Raphael Joye, Vladimir L. Cousin, Iliona Malaspinas, Leonce Mwizerwa, Maya Bouhabib, Tomasz Nalecz, Tornike Sologashvili, Maurice Beghetti, Arnaud G. L’Huillier, Julie Wacker

**Affiliations:** 1 Pediatric Cardiology Unit, Department of Woman, Child, and Adolescent Medicine, Faculty of Medicine, Geneva University Hospitals, 1205 Geneva, Switzerland; vladimir.cousin@hcuge.ch (V.L.C.); iliona.malaspinas28@gmail.com (I.M.); leonce.mwizerwa@hcuge.ch (L.M.); mayasabrina.bouhabib@hcuge.ch (M.B.); julie.wacker@hcuge.ch (J.W.); 2 Pediatric Intensive Care Unit, Department of Woman, Child, and Adolescent Medicine, Faculty of Medicine, Geneva University Hospitals, 1205 Geneva, Switzerland; 3 Pediatric Cardiac Surgery Unit, Department of Surgery, Faculty of Medicine, Geneva University Hospitals, 1205 Geneva, Switzerland; tomasz.nalecz@hcuge.ch (T.N.); tornike.sologashvili@hcuge.ch (T.S.); 4 Pediatric Infectious Disease Unit, Department of Woman, Child, and Adolescent Medicine, Faculty of Medicine, Geneva University Hospitals, 1205 Geneva, Switzerland; arnaud.lhuillier@hcuge.ch

**Keywords:** infective endocarditis, invasive infection, *Kingella kingae*, children, cardiac imaging, cardiac surgery

## Abstract

Infective endocarditis due to *Kingella kingae* is a rare but serious invasive infection that occurs mostly in children. Recent advances in nucleic acid amplification testing as well as in cardiac imaging have enabled more accurate diagnosis. A good understanding of the epidemiology and virulence factors remains crucial to guide the therapeutic approach. Here, we synthesize the current state of knowledge on epidemiological features, pathophysiological insights, complications, and therapy regarding *Kingella kingae* endocarditis in children and adults. Finally, throughout this comprehensive review, knowledge gaps and areas for future research are also identified.

## 1. Introduction

*Kingella kingae (K. kingae)* is a facultative anaerobic, β-hemolytic, encapsulated organism belonging to the *Neisseriaceae* family of Gram-negative bacteria that yields positive oxidase and negative catalase reactions [1,2,3]. Since the early 1990s, advances in culture and molecular diagnostic methods have significantly improved *K. kingae* detection, leading to its recognition as an important cause of invasive infection in children [4,5,6]. Epidemiological studies have shown that *K. kingae* belongs to the commensal flora of the oropharynx of healthy children aged 6 months to 4 years and is carried by 10–12% of children aged 12 to 24 months [5,6,7]. While most children with *K. kingae* pharyngeal carriage remain asymptomatic, the reported annual incidence of invasive infections reaches 9.4 cases per 100,000 children [8]. Although *K. kingae* is widely recognized as a frequent cause of pediatric osteoarticular infections and bacteremia, it has also been associated with pneumonia, meningitis, peritonitis, and infective endocarditis (IE) [4,9,10]. Infective endocarditis is a major clinical challenge associated with high mortality and morbidity [11,12]. In adults, the estimated incidence of IE is 13.8/100,000 subjects per year [11,13]. In the pediatric population, the reported incidence is lower, ranging from 0.45 to 0.84/100,000 subjects per year, but with an upward trend being observed in recent years [12,14,15,16,17,18]. *K. kingae* is a rare cause of IE typically affecting children and is associated with a high rate of complications [19,20].

In this article, we sought to review the current state of knowledge on the physiopathological, epidemiological, and clinical features of IE due to *K. kingae*, as well as discuss diagnostic issues and treatment strategies.

## 2. Pathogenicity

Invasive infections including IE require several pathophysiological steps. First, *K. kingae* must colonize the host’s oropharynx, and therefore adhere to the respiratory epithelium. Afterward, a breach in the epithelial barrier is required to allow the organism to enter the bloodstream where dissemination to distant sites can take place. Several pathogenic characteristics of *K. kingae* such as pili, exotoxin, polysaccharide capsule, and galactan exopolysaccharide seem to be essential for the bacteria to survive in the intravascular compartment as well as in various body sites such as endocardium [21], and these are detailed hereafter.

Adherence and motility are mainly mediated by the type 4 pili (T4P) and a trimeric autotransporter named Kingella Nhha homolog (Knh). The T4P are filamentous surface fibers that are of paramount importance for various virulence factors such as surface twitching motility, adherence to human cells, and competence for transformation, that enables the uptake of exogenous DNA [6,21,22]. The *PilA1* gene encodes for the major pilin subunit and is therefore needed for T4P genesis [22]. Furthermore, the level of piliation has been shown to vary among various *K. kingella* strains and also within a single strain subculture as well [22]. The piliated strains exhibit a high level of adherence to the epithelial and synovial cells. Interestingly, bacteria isolated from patients with IE are non-piliated, while the strains associated with bacteriemia without a focal infection exhibit T4P. This suggests that piliation is not required to invade endocardium [6,22]. Moreover, adherence is significantly enhanced by Knh, which has been shown to exhibit a stronger adhesive effect than T4P when considered separately and under shear stress [21,22,23]. In addition to adherence, T4P filaments are also capable of retraction, an ability necessary for twitching motility and natural competence. Two cytoplasmic enzymes (PilT and PilU) remove PilA1 subunits from the base of the filaments, enabling T4P dynamic retraction [23].

Once the respiratory epithelium is colonized, translocation across the epithelium is mediated, among other factors, by the RTX toxin, which belongs to the family of pore-forming exotoxins [21]. The RTX toxin is encoded by the *rtx* gene found in all strains of *K. kingae,* as well as in *Kingella negevensis* species [24]. Additionally to *rtx*, four other genes (*rtxB*, *rtxC*, *rtxD*, and *tolC*) are required for production, activation, and exocytosis of the RTX toxin and are located on two loci that are thought to have been acquired through horizontal transfer from other bacterial species [25,26]. RTX toxin is synthetized in a non-toxic form that needs further acyltransferase-mediated activation by the RtxC protein [27,28]. After activation, RTX toxin is secreted through an outer membrane protein encoded by *rtxB*, *rtxD,* and *tolC* [29]. Its cytotoxic effect is then mediated through insertion and pore formation into cell membranes, leading to cell death [21]. Data issued from a rat model have provided important insight on RTX toxin’s role during invasive infection. The inoculation of a *K. kingae* strain isolated from a child with septic arthritis led to a dramatic decrease in white blood cell count, multiple organ failure, and death. Meanwhile, animals inoculated with an RTX toxin-deficient mutant of the same strain did not show any sign of disease [30].

Moreover, RTX toxin can also be secreted via outer membrane vesicles. These are small, spherical structures, derived from the bacterial membrane, which allow Gram-negative bacteria to release various toxins and proteins [6,21]. These vesicles are typically phagocytosed by many eukaryote cells distant from the bacteria, promoting a cytotoxic effect and an increased inflammatory response, thus potentializing RTX toxin’s virulence [31].

Protection from phagocytosis, as well as neutrophils and complement-mediated killing, is typically provided by a polysaccharide capsule in many human pathogens [6]. In fact, *K. kingae’s* lipid-anchored polysaccharide capsule has been visualized using thin-section transmission electron microscopy after being stained with cationic ferritin [23]. The genetic basis of *K. kingae* encapsulation has been widely studied; findings suggest that capsule genes may be scattered throughout the genome [32]. An ABC-transporter-type operon called *ctrABCD*, which shares a homologous locus in *Neisseria meningitidis* genome, is required for capsule export and surface presentation [23]. Furthermore, two other genes, *LipA* and *LipB*, are essential for capsular surface localization [32]. Similarly, homologs of these genes found in *Escherichia coli* and *Neisseria meningitidis* were shown to mediate the linking between the lipid membrane and the polysaccharide capsule [33]. Finally, the capsule synthase, encoded by the *csaA* gene, is necessary to generate the [3)-β-Gal*p*NAc-(1→5)-β-Kdo*p*(2→] polysaccharide capsule (type A) [32]. Further investigation revealed that four distinct synthases encoded by four genetic loci (*csa, csb, csc,* and *csd*) define the capsule type (A, B, C, and D, respectively) [34]. The different types are characterized according to their molecular structure as follows: [6)-α-Glc*p*NAc-(1→5)-β-(8-OAc)Kdo*p*-(2→] for type B, [3)-β-Rib*f*-(1→2)-β-Ribf-(1→2)-β-Rib*f*-(1→4)-β-Kdo*p*-(2→] for type C, and [P-(O→3)[β-Gal*p*-(1→4)]-β-Glc*p*NAc-(1→3)-α-Glc*p*NAc-1-] for type D [33,34]. Interestingly, these four types seem to be key components of virulence factors among *K. kingae* strains [33]. In a French cohort, type C and D capsules were mainly encountered in strains colonizing the oropharynx of healthy children, while types A and B appeared to have a higher pathogenic profile [35]. Nevertheless, these observations were made mainly on osteoarticular infections, and further studies are needed to better characterize their role in the pathogenicity of endocarditis. As described above, adherence is a critical determinant for colonization of the respiratory epithelium; an interplay between T4P, Knh, and the polysaccharide capsule is mandatory for full-level adherence [23,36]. First, T4P interacts with the host respiratory epithelium cell. Through PilT-mediated retraction, the bacteria are then pulled toward the host cell membrane, physically displacing the polysaccharide capsule and exposing Knh to the respiratory epithelium [23]. This process therefore enables Knh-mediated high-affinity adherence without the necessity of genetic capsule downregulation [23]. Furthermore, it has been proven that the polysaccharide capsule was of paramount importance in evading the host immune response. Data from a rat model highlighted the critical role of *K. kingae* polysaccharides in preventing opsonin (IgG, IgM, C4b, and C3b) deposition and complement-mediated killing, thus improving intravascular survival and enhancing virulence [37]. Moreover, an in vitro study recently showed that *K. kingae’s* polysaccharide capsule is able to prevent neutrophil activation and binding, as well as the production of reactive oxygen species [38].

In addition to the lipid-anchored polysaccharides, *K. kingae* also exhibits the ability of galactan exopolysaccharide secretion, which is involved in biofilm establishment, growth, and architectural remodeling [6]. This expolysaccharide is a polymer of galactofuranose that is present in two recognized structures in *K. kingae*, based on the link connecting the galactofuranose residues [39]. Interestingly, data from in vivo studies have shown that the galactan exopolysaccharide shares an overlapping effect with the polysaccharide capsule in terms of resistance to opsonization [37]. Additionally, the galactan exopolysaccharide exhibits a distinct function from polysaccharides when it comes to neutrophil evasion, such as reducing neutrophil phagocytosis and sensitivity to antimicrobial peptides [38].

Several genotyping studies using various molecular methods have sought to describe the global genetic diversity of *K. kingae* species and to determine which strains are responsible for invasive infections. Multilocus sequence typing, pulsed-field gel electrophoresis, as well as single-gene typing methods targeting the *rtxA* or the *por* genes have identified 40 sequence types, 18 *rtxA* alleles, and 12 *por* alleles [40,41,42,43]. Furthermore, one specific sequence type, named ST-24, seemed to be significantly associated with cases of IE [40]. More recently, an analysis performed by a more rapid and cost-effective molecular typing tool targeting the DNA uptake sequence revealed that only five sequence-type complexes accounted for the majority of *K. kingella* strains found worldwide. Moreover, this study confirmed that most IE cases were due to ST-24 [43]. Finally, a recent study analyzed the whole genomes of 125 international *K. kingae* strains isolated in healthy carriers and patients with invasive infections. While the study failed to identify single genes able to discriminate between colonizers and invasive organisms, significant differences in the distribution of multiple genes were found among them. Interestingly, a gene encoding the iron-regulated protein FrpC was absent in all strains associated with IE, suggesting a potential role in endocarditis pathogenicity [44]. However, data on the relationship between serotypes and the severity of endocarditis remain scarce, and further studies are needed to better understand the specificities of endocarditis.

## 3. Infective Endocarditis

### 3.1. Epidemiology and Clinical Features

While *K. kingae* asymptomatic carriage and osteoarticular infections are well known, data on IE epidemiology remain scarce. Colonization of an infant’s oropharynx typically begins around 6 months, which probably corresponds to the decline in maternal antibodies and the onset of daycare attendance [2,45,46]. The colonization rate peaks in children aged 12–24 months, with a reported prevalence ranging from 9 to 23% depending on study populations, and decreases afterwards [7,47,48,49]. A single subject is generally subsequently colonized by several different strains, each lasting weeks to months, thus indicating that a strain-specific immune response is triggered by carriage without the need for invasive infections [2,47]. Moreover, it is important to keep in mind that less than 1% of asymptomatic pediatric carriers will develop an invasive infection [7].

Most invasive infections due to *K. kingae* are osteoarticular and caused by a breach through the respiratory epithelium, allowing the bacteria to invade the bloodstream. Therefore, the incidence of *K. kingae* invasive infections increases during autumn and winter, mirroring respiratory viral infections such as rhinovirus and enterovirus (hand foot mouth), as well as HSV gingivostomatitis [6,50]. The annual incidence of invasive infection was estimated at 9.4 per 100,000 children <4 years old in a study conducted in southern Israel [6]. This incidence is very likely underestimated, as it is based on insensitive culture detection methods as discussed below. Among 143 children with culture-proven *K. kingae* invasive disease, the highest number of infections was reported between the ages of 6 and 17 months [6]. *K. kingae* belongs to the HACEK group (*Haemophilus* species, *Aggregatibacter* specie*s*, *Cardiobacterium hominis*, *Eikenella corrodens*, and *Kingella* species) responsible for 3–5% of all IE cases [6], with the majority of these reported in children and infants [1]. We reviewed the literature and identified 32 articles describing 45 children diagnosed with *K. kingae* IE [8,19,51,52,53,54,55,56,57,58,59,60,61,62,63,64,65,66,67,68,69,70,71,72,73,74,75,76]. While osteoarticular infections mostly occur in children <4 years, 22% (10/45) of IE occurred in children >4 years. Fifty one percent of cases (23/45) had a history of congenital heart disease or rheumatic valvular disease. A native valve was affected in 96% (43/45) and a prosthetic one in only 4%. Most infections were left-sided IE, with the mitral valve being the most frequently affected. In contrast to other invasive infections caused by *K. kingae*, children with IE typically exhibit high fever, elevated white blood cell counts, and inflammatory biomarkers such as C-reactive protein, as well as an elevated erythrocyte sedimentation rate. Furthermore, a high rate of complications was reported (67%, 30/45), such as valvular perforation/severe regurgitation (13), heart failure/cardiogenic shock (2), thromboembolic events (7), meningitis/brain abscess (9), stroke (13), and death (4). Table 1 describes the characteristics of IE cases caused by *K. kingae.*

In the adult population, data are scarce. We identified less than 20 patients with *K. kingae* IE reported in the literature (Table 1) [77,78,79,80,81,82]. The median age was 56 years (IQR 41–63) and 39% of the patients were male. Around 20% (4/18) of these patients had a history of immunosuppression. Fever and elevated inflammatory biomarkers were present in all the patients. While, similarly to the pediatric cases, all the infections reported involved the aortic valve or the mitral valve, around 44% (8/18) of the infections involved a prosthetic valve. We identified fewer complications than in the pediatric population (28%, 5/18), including heart failure/cardiogenic shock (4), stroke (1), and death (1).

Of note, the incidence of *K. kingae* IE may well be underestimated; many cases could have been classified as culture-negative endocarditis, as discussed below.

### 3.2. Diagnosis

Infective endocarditis is usually suspected in the presence of fever without source and positive blood culture in children or adults with one or more risk factors (i.e., valvular disease, congenital heart disease, implantable electronic devices, central venous catheter, and immunosuppression). The modified Duke criteria rely on the detection of four phenomena that are involved in IE pathophysiology, namely bacteremia, valvular involvement, immunologic, and embolic processes. Therefore, the use of these criteria in the diagnostic process of IE is recommended in adults and children [11,12,83]. In addition, the recently updated European guidelines for the management of infective endocarditis in adults proposed further improvements to these criteria to overcome some of their limitations (Supplementary Material Table S1) [11].

The identification of *K. kingae* from two separate blood cultures is a major criterion for IE diagnosis [11]. Yet, as mentioned above, organisms belonging to the HACEK group, including *K. kingae*, are fastidious Gram-negative bacilli that grow slowly and therefore are difficult to identify using traditional cultures on solid media [2]. A study published in the early 1990s showed that the inoculation of blood and joint fluid into BACTEC 460 aerobic blood culture bottles improved the identification of *K. kingae* by diluting detrimental components [84]. However, the identification of *K. kingae* remained suboptimal, with many cases of IE considered as culture-negative [2,11]. Around 10 years later, studies on nucleic acid amplification methods found significantly enhanced *K. kingae* detection rates in bone and joint infections as well as in IE [2]. The initial approach relied on a broad range polymerase chain reaction (PCR) targeting the gene coding for 16S ribosomal RNA This enabled the detection of DNA from various fastidious bacteria, including *K. kingae* in 23% of cases with culture-negative infections [85]. Throughout the years, this method has gradually been replaced by *K. kingae*-specific real-time PCR tests targeting the *rtx* gene, the chaperoin 60 gene, and the malate dehydrogenase gene [86,87]. These methods showed improved sensitivity compared to 16S PCR. However, targeting the *rtx* locus does not allow us to distinguish between *K. kingae* and *Kingella negevensis*, thus the approach lacks specificity. Furthermore, primers from the chaperoin 60 gene-based PCR assays showed suboptimal sensitivity. Targeting the malate dehydrogenase gene has achieved very high specificity and sensitivity and should therefore be the preferred method, enabling a detection rate four times higher than that of blood culture. [87]. Finally, it is strongly recommended that tissue or prosthetic material obtained during surgery for endocarditis undergoes cultures and broad-range PCR, especially in the case of culture-negative IE. As the bacterial load in the blood is significantly lower than on affected tissues, the sensitivity of PCR and cultures on endocardium or prosthetic devices is enhanced [11].

**Figure 1:**
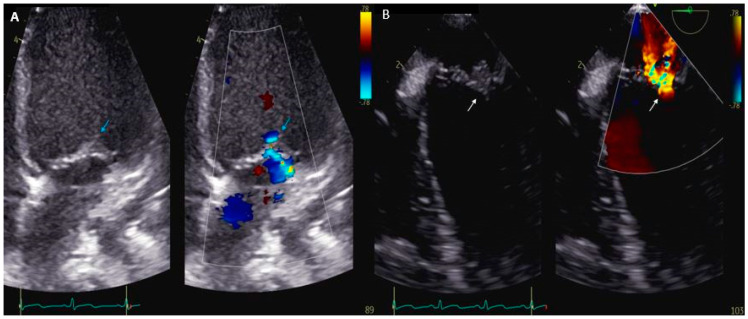
(**A**) Transthoracic echocardiography of a 5-year-old girl with infective endocarditis of the mitral valve with mild regurgitation (blue arrow). (**B**) Transesophageal echocardiography of the same patient that enables a better visualization of the vegetation on the anterior leaflet, as well as a better characterization of the regurgitation (white arrow).

**Figure 2:**
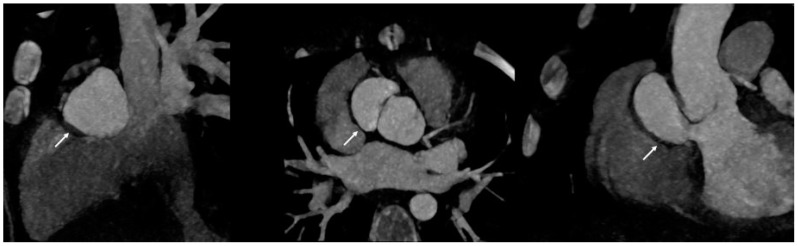
Cardiac CT of a 6-year-old girl with infective endocarditis due to *Kingella kingae* complicated by a paraaortic pseudoaneurysm (white arrow).

**Figure 3:**
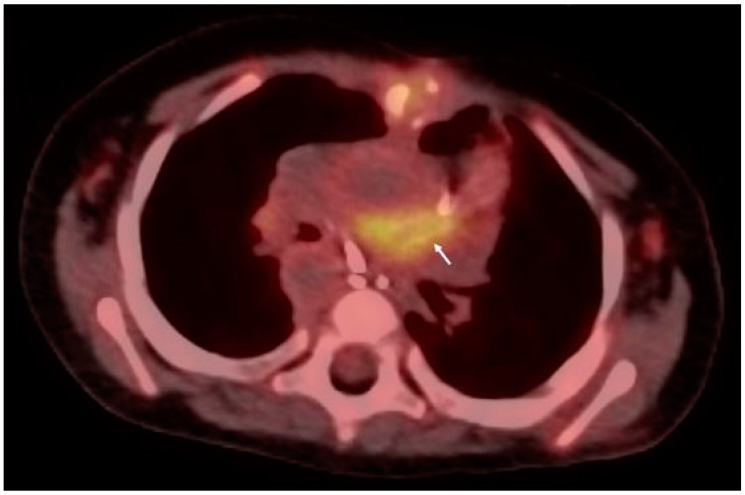
^18^F-fluorodeoxyglucose positron emission tomography of a 19-month-old boy with infective endocarditis of a Sano conduit between the right ventricle and the pulmonary artery (white arrow).

Evidence of endocardial involvement is the other major diagnostic criterion. Therefore, cardiac imaging is one cornerstone of IE diagnosis, even though it is not specific to *K. kingae.* Echocardiography is the first-line imaging technique when evaluating IE on a native or prosthetic valve. Transthoracic echocardiography (TTE) is widely accessible, cost-effective, and requires no sedation. However, despite a specificity of 94% in detecting vegetation on native or prosthetic valves, its sensitivity of around 60% is mediocre in adult studies [88]. Therefore, transesophageal echocardiography (TEE) is mandatory in all adults with suspected IE [11]. Conversely, TTE alone may be sufficient for the visualization of vegetations in children weighting <60 kg with satisfactory acoustic windows, in most cases [12]. Echocardiography findings encompass vegetation’s characteristics and size, valvular regurgitation or stenosis, perivalvular abscesses, and leaflet perforation or intracardiac fistula [11,88] (Figure 1). A normal TTE or TEE cannot rule out the diagnosis of endocarditis. In the case of negative or inconclusive examination and high clinical suspicion, repeating TTE and/or TEE is recommended within 5–7 days [89]. Moreover, echocardiographic reassessment is also indicated after IE diagnosis confirmation when a new complication is suspected, or before switching to oral antibiotic therapy [11,90]. Cardiac computed tomography (CT) might be helpful in the detection of perivalvular complications (Figure 2). A meta-analysis published in 2021 found that cardiac CT performed better than TEE for detecting prosthetic valve IE (specificity 94%, sensitivity 78%), as well as periannular affections (specificity 93%, sensitivity 88%) [91]. Another study published similar results for paravalvular abscesses and pseudoaneurysms [92]. Moreover, as opposed to CT, the role of cardiac magnetic resonance imaging (MRI) is limited to identifying valvular and paravalvular lesions due to lower spatial resolution. However, MRI displays an excellent sensitivity for detecting neurological lesions [11]. Cerebral MRI therefore remains a crucial examination when evaluating the extent of embolic phenomena in patients with IE, especially when mitral or aortic valves are involved [93]. In fact, up to 80% of those patients may show signs of subclinical embolization on brain MRI, which could help in navigating the complex decision about surgical intervention in IE [94]. Finally, the place that nuclear imaging holds in IE diagnosis is growing. ^18^F-fluorodeoxyglucose-positron emission tomography (^18^F-FDG-PET) CT provides functional information based on the increased glucose uptake and glycolysis of metabolically active cells. In adults with a native valve, the specificity of ^18^F-FDG-PET CT is very high (around 98%) for valvular and paravalvular endocarditis, but its sensitivity is mediocre (30%) [95]. For prosthetic valve endocarditis, the estimated sensitivity increases at 86% with an unchanged specificity, making ^18^F-FDG-PET CT the examination of choice in the case of inconclusive TTE/TEE [11,96]. Furthermore, in children and young adults with congenital heart disease, ^18^F-FDG-PET CT has been reported to be accurate in detecting and localizing IE either before or after cardiac surgery (Figure 3) [97]. It was previously recommended to perform ^18^F-FDG-PET CT at least 3 months after cardiac surgery to avoid misinterpretation of “normal” FDG uptake, especially after prosthetic valve implantation [11,12]. Yet, recent data support the notion that analysis of FDG uptake patterns could be sufficient to differentiate postoperative inflammatory changes from infection, even after recent valve surgery, thus questioning the recommended 3-month period [98].

### 3.3. Treatment

The recommendations for antimicrobial therapy for IE are similar for children and adults. First, if the patient is not critically ill, antibiotic administration may reasonably be delayed for ≥48 h while additional blood cultures are obtained [11,12]. After the identification of *K. kingae*, the recommended antibiotic therapy is a 4-week course for native valve endocarditis and a 6-week course for prosthetic valve endocarditis [11,83]. Most societies recommend ceftriaxone or another third-generation cephalosporin as a first-line therapy, given the fact that some strains produce beta-lactamases [11,12,83]. Ten percent of the *K. kingae* colonizing strains found in asymptomatic subjects and less than two percent of invasive strains produce beta-lactamase. It is therefore mandatory to test any isolated *K. kingae* for beta-lactamase production [99]. If the identified strain does not produce beta-lactamases, ampicillin can be used as an alternative. However, the opinions of societies differ on whether gentamicin should be associated to ampicillin or not [83], and if so, whether it should be for the first two weeks [11] or for the whole course [12]. Even though the evidence is limited, ciprofloxacin is also proposed as an alternative [11,83].

Furthermore, *K. kingae* is resistant to glycopeptides and clindamycin. This is a serious consideration when patients with *K. kingae* endocarditis are empirically administered vancomycin to cover potential methicillin-resistant *Staphylococcus aureus*, before cultures or nucleic amplification results are ready [100]. Finally, *K. kingae* is almost always susceptible to aminoglycosides, macrolides, tetracycline, and co-trimoxazole [100].

Infective endocarditis is sometimes associated with complications that can only be addressed by cardiac surgery. Currently, recommendations for the surgical management of pediatric IE are mostly based on adult studies. The principal reasons for surgery in the setting of an acute IE are heart failure/severe valve dysfunction, uncontrolled infection, and septic embolic phenomena, especially to the central nervous system [11,12]. Several algorithms are proposed for particular cases, such as IE in a patient with congenital heart disease, prosthetic valve endocarditis, as well as IE due to specific organisms, but those are beyond the scope of this article [11]. Regarding *K. kingae*, there is no consensus on the optimal timing for surgery. *K. kingae* IE is associated with a high risk of neurological complications, mostly stroke and bleeding. Lenoir et al. reported their experience with two children treated for *K. kingae* endocarditis. In patients with neurological complications, they recommend delaying cardiac surgery in order to reduce the risk of secondary cerebral hemorrhage or infarction following cardiopulmonary bypass, and to facilitate valve repair when endocarditis is no longer active [75]. Furthermore, early surgery, especially when dealing with a large vegetation on the left side, can improve neurological prognosis and allow retention of the native valve [64]. It is indeed of paramount importance to aim for valve preservation without foreign material implantation and to minimize the risk for subsequent surgery, especially in children [64,75]. Further studies are needed to determine the best surgical management timings and strategies.

## 4. Conclusions

Infective endocarditis due to *K. kingae* is a rare though critical invasive infection that occurs mostly in children. Recent advances in nucleic acid amplification testing have enabled more accurate and rapid diagnosis. Many cases that would have otherwise been classified as culture-negative endocarditis are likely to be identified as *K. kingae* IE, which will probably enrich the data available on this infection. Finally, while the antibiotic susceptibility of *K. kingae*, as well as its antimicrobial treatment are well known, more data are needed to better define the role and timing of surgical procedures and predict outcomes in children and adults.

## Figures and Tables

**Table 1:** Characteristics of infective endocarditis cases caused by *Kingella kingae*.

	Children (n = 45)	Adults (n = 18)
Sex, n (%)		
Female	13 (29)	6 (33)
Male	20 (44)	7 (39)
Unknown	12 (27)	5 (28)
Age, median (IQR)	19 months (14–48)	56 years (41–63)
Previous heart disease, n (%)	23 (51)	8 (44)
Congenital heart disease	21 (47)	5 (28)
Rheumatic heart disease	2 (4)	3 (17)
None	22 (49)	3 (17)
Unknown	2 (4)	7 (39)
Endocarditis localization, n (%)		
Mitral valve	26 (58)	7 (39)
Aortic valve	4 (9)	7 (39)
Tricuspid valve	1 (2)	0 (0)
Pulmonary valve	1 (2)	0 (0)
Other	2 (4)	0 (0)
Unknown	11 (25)	6 (33)
Type of valve, n (%)		
Native	43 (96)	4 (22)
Prosthetic	1 (2)	8 (44)
Unknown	1 (2)	6 (33)
Complications, n (%)		
Valvular perforation/severe regurgitation	13 (29)	0 (0)
Paravalvular abscess/pseudoaneurysm	2 (4)	1 (6)
Heart failure/cardiogenic shock	2 (4)	4 (22)
Pulmonary embolism	1 (2)	0 (0)
Systemic embolism (other than cerebral)	6 (13)	0 (0)
Meningitis/brain abscess	9 (20)	0 (0)
Stroke	13 (29)	1 (6)
Death	4 (9)	1 (6)
None	7 (16)	6 (33)
Unknown	9 (20)	5 (27)

## Data Availability

No new data were created or analyzed in this study. Data sharing is not applicable to this article.

## Supplementary Materials

The following supporting information can be downloaded at: https://www.mdpi.com/article/10.3390/microorganisms12010164/s1, Table S1: Comparison of 2015 (children) and 2023 (adults) diagnostic criteria for infective endocarditis.
