# Supplementary material for: Infective Endocarditis Due to Kingella kingae
Source: Microorganisms. 2024 Jan 13;12(1):164. doi: 10.3390/microorganisms12010164 (PMC10819173; doi:10.3390/microorganisms12010164)
Supplement: Supplementary file 1 [file microorganisms-12-00164-s001.zip › microorganisms-2769403-supplementary.pdf]

Supplemental Digital Content

Table S1. Comparison of 2015 (children) and 2023 (adults) diagnostic criteria for infective endocarditis. Shaded cells indicate that no modifications have been made between the two versions.IE: infective endocarditis, HACEK: *Haemophilus, Aggregatibacter, Cardiobacterium, Eikenella, and Kingella*, TEE: transesophageal echocardiography, TTE: transthoracic echocardiography, CT: computed tomography, [18F]-FDG-PET/CT: ^{18}F-fluorodeoxyglucose positron emission tomography, WBC SPECT/CT: white blood cell single photon emission tomography.

**Modified Duke Criteria (2015 AHA Guideline for Management of Infective Endocarditis in Children and Adults)**	**2023 European Society of Cardiology modified diagnostic criteria**
**Major critera**	
1. Positive blood culture for IE	
A. Typical microorganism consistent with IE from ≥2 blood cultures, as noted below	Typical microorganisms consistent with IE from two separate blood cultures:<br>- Oral streptococci<br>- Streptococcus gallolyticus (formerly S. bovis)<br>- HACEK group<br>- S. aureus<br>- E. faecalis (acknowledged as a typical endocarditis bacterium)
(I) Viridans streptococci, *Streptococcus bovis*, or HACEK group	
(II) Community-acquired Staphylococcus aureus or enterococci, in the absence of a primary focus	
B. Microorganisms consistent with IE from persistently positive blood cultures, defined as:	
(I) ≥2 Positive cultures of blood samples drawn >12 hours apart	
(II) All of 3 or a majority of ≥4 blood cultures, (with first and last samples drawn ≥1 hour apart).	
(III) Single positive blood culture for *Coxiella burnetii* or antiphase-I immunoglobulin G antibody titer >1:800	
2. Evidence of endocardial involvement/Imaging positive for IE	
A. Positive echocardiogram (TEE recommended in prosthetic valves, rated at least possible IE by clinical criteria, or complicated IE; TTE as the first test in other patients) for IE, defined as	Valvular, perivalvular/periprosthetic and foreign material anatomic and metabolic lesions characteristic of IE detected by any of the following imaging techniques:<br>- Echocardiography (TTE and TEE).<br>- Cardiac CT.<br>- [18F]-FDG-PET/CT.<br>- WBC SPECT/CT.
(I) Oscillating intracardiac mass on valve or supporting structures, in the path of regurgitant jets, or on implanted material in the absence of an alternative anatomic explanation<br>or	
(II) Abscess	

or	
(III) New partial dehiscence of prosthetic valve<br>or	
B. New valvular regurgitation (worsening or changing of preexisting murmur not sufficient)	
**Minor citeria**	
1. Predisposition: predisposing heart condition or IV drug use	
2. Fever: temperature ≥38.0°C	
3. Vascular phenomena: major arterial emboli, septic pulmonary infarcts, mycotic aneurysm, intracranial hemorrhage, conjunctival hemorrhages, and Janeway lesions	
4. Immunologic phenomena: glomerulonephritis, Osler nodes, Roth's spots, and rheumatoid factor	
5. Microbiological evidence: positive blood culture but does not meet a major criterion as noted above or serological evidence of active infection with organism consistent with IE	
**IE Classification (at admission and during follow-up)**	
Definite:<br>- 2 major criteria.<br>- 1 major criterion and at least 3 minor criteria.<br>- 5 minor criteria.	
Possible:<br>- 1 major criterion and 1 or 2 minor criteria.<br>- 3–4 minor criteria.	
Rejected:<br>- Does not meet criteria for definite or possible at admission with or without a firm alternative diagnosis.	
